# Anti-bacterial Efficacy of Zirconium Oxide Nanoparticles on Streptococcus mutans and Enterococcus faecalis: An In Vitro Study

**DOI:** 10.7759/cureus.75421

**Published:** 2024-12-09

**Authors:** Anu Priya Guruswamy Pandian, Anil Kumar Ramachandran, Priyanka Kodaganallur Pitchumani, Blessy Mathai, Davis C Thomas

**Affiliations:** 1 Endodontics, Private Practice, Los Angeles, USA; 2 Endodontics, Ragas Dental College and Hospital, Chennai, IND; 3 Periodontics, The University of Iowa College of Dentistry and Dental Clinics, Iowa City, USA; 4 General Dentistry, Private Practice, Newark, USA; 5 Orofacial Pain, Eastman Institute for Oral Health, Rochester, USA; 6 Diagnostic Sciences, Rutgers School of Dental Medicine, Newark, USA

**Keywords:** anti-microbial susceptibility testing, dental caries, enterococcus faecalis, nanoparticles, secondary endodontic infections, streptococcus mutans, zirconium oxide

## Abstract

Introduction

Complex interactions between cariogenic bacteria and host factors modulate dental caries. *Streptococcus mutans*, a gram-positive facultative anaerobe plays a prominent role in the initiation of caries. The ability of *S. mutans* to adhere to salivary enamel pellicle results in an acidic local habitat for the organism. This leads to demineralization of the tooth and penetration of bacteria into the pulp leading to endodontic infections. *Enterococcus faecalis, *an opportunistic pathogenis a gram-positive, facultative anaerobe implicated in secondary endodontic infections. This study aimed to evaluate the anti-bacterial efficacy of zirconium oxide nanoparticles (ZrO_2_ NPs) against *S. mutans *and *E. faecalis. *

Materials and methods

Standard *S. mutans *and *E. faecalis* strains were subcultured at specific temperatures for 24 hours. *S.mutans* was subcultured onto blood agar and colonies of *E. faecalis* were cultured on nutrient agar. The strains were tested for their sensitivity to ZrO_2_ NPs at various dilutions. The standard methods determined the minimum concentration of ZrO_2 _NPs to inhibit 99.9% growth of *S. mutans* and *E. faecalis*.

Results

The zones of inhibition were compared with gentamicin as a control. ZrO_2_ NPs exhibited clear zones of inhibition of 12 mm and 15 mm at 100 mg/mL concentrations against *S. mutans* and *E. faecalis* in the agar wells, respectively.

Conclusion

The present study concluded that ZrO_2_ NPs have potential anti-bacterial activity against both *S. mutans *and *E. faecalis*.

## Introduction

Dental caries is modulated by complex interactions between cariogenic bacteria and host factors [1]. *Streptococcus mutans*, a gram-positive facultative anaerobe, plays a prominent role in the initiation of caries [2,3]. The ability of *S. mutans* to adhere to salivary enamel pellicle results in an acidic local habitat for the organism. This leads to demineralization of the tooth and penetration of bacteria into the pulp leading to endodontic infections [4]. While primary endodontic infections are dominated by gram-negative anaerobes, secondary infections seem to be predominantly caused by gram-positive facultative anaerobes such as *E. faecalis* [5,6]. The pathogenic role of *E. faecalis *in repeated endodontic treatment failures can be attributed to its ability to invade and colonize the dentinal tubules under stressful conditions such as nutrient deficiency [7]. These organisms grow in chains of cells within the dentinal tubules and remain viable, surviving chemo-mechanical instrumentation, and intracanal medications. This facilitates re-infecting the obturated root canals. Resistance to antimicrobial agents and intracanal disinfection procedures, and enduring periods of starvation result in a high prevalence of *E. faecalis* in secondary endodontic infections [8,9]. Owing to its high survival rate within the root canal, we chose* E. faecalis* as our organism, along with *S. mutans* for the present study.

The complex anatomy and subsequent access issues make the elimination of bacterial biofilms a prime challenge in endodontics [10]. Although acceptable success is achieved in approximately 80% of performed endodontic procedures, there is a real need for more efficient disinfection and antimicrobial strategies [11]. Ongoing research has focused on newer nanoparticles (NPs) that may be efficacious against endodontic microbes [12].

Among nanostructured materials, metal oxide NPs, and metal complexes are being studied in medicine. These highly ionic metal oxides, in addition to their wide range of desirable physical and chemical properties, also possess antibacterial activity [13]. One of the metal oxides that is extensively used for their mechanical properties in dentistry is zirconium oxide (ZrO_2_). However, studies that evaluated its antimicrobial efficacy are minimal. Previously, the antimicrobial activity of ZrO_2_ NPs against a few bacterial and fungal strains has been shown [13-15]. We could not find studies that evaluated the antibacterial efficacy of ZrO_2_ NPs against *S. mutans* and *E. faecalis*. Hence, the purpose of this study was to evaluate the minimum inhibitory concentration (MIC) of ZrO_2_ NPs against *S. mutans* and *E. faecalis*.

## Materials and methods

This was an in vitro study conducted from February 5, 2018, to September 28, 2018. The microbial studies were performed at Ragas Dental College and Hospital, Chennai, Tamil Nadu, India. The scanning electron microscopy (SEM) and energy dispersive X-ray (EDX) analysis to determine particle size and purity was performed at the Indian Institute of Technology (IIT), Madras, Chennai.

ZrO_2 _NPs of particle sizes ranging from 15-20 nm in diameter were obtained from a reputed professional laboratory, Nano Research Lab, Jharkhand, India. The ZrO_2_ NPs were of 99.9% purity, with a specific surface area of 40-45 m^2^/g. The melting point of the nanoparticles was 2715^o^C. The ZrO_2_ NPs also contained trace levels of aluminum (less than 0.06%), lead, and iron (less than 0.02% respectively). The particle size and purity of ZrO_2_ NPs were re-confirmed with high-resolution scanning electron microscopy and energy-dispersive X-ray spectroscopy. Strains of *E. faecalis* (ATCC 29212) and *S. mutans* (ATCC 25175) were obtained from Sigma Aldrich Chemicals Private Ltd. (Bengaluru, India) and subcultured onto brain heart infusion agar plate (Ref. MV 210-500G; HiMedia Laboratories Private Limited, Maharashtra, India) at 37^o^C for 24 hours.

Anti-microbial susceptibility testing of ZrO_2_ nanoparticles against *E. faecalis* and *S. mutans*

Agar diffusion assay is the most common technique for assessing microbial susceptibility to antibiotics, and it has a number of variations of which well- and disc-diffusion methods are the qualitative indicators standardized by the Clinical and Laboratory Standards Institute (CLSI) for antibiotic testing [16].

The agar diffusion method is commonly used for the determination of MIC in solid media that involves the application of different concentrations of antibiotic solutions (ZrO_2_ NPs) punched into agar plates seeded with the test bacterial strain (*S. mutans *and *E. faecalis*). The diffusion of antibiotics from these wells into the agarose medium leads to the inhibition of bacterial growth in the vicinity of the source, leading to the formation of clear ’zones’ that have no bacterial growth [17].

The antimicrobial activity of the ZrO_2_ NPs against strains of *E. faecalis* and* S. mutans* was performed by agar well diffusion method [18-20]. The test cultures were inoculated into nutrient broth, incubated at 37^0^C for 24 hours, and then transferred onto Muller-Hinton agar plates using sterile swabs. Stock solutions of ZrO_2 _NPs with a concentration of 100 mg/ml were prepared in deionized water. Since nanoparticles form aggregates, the stock dispersion was sonicated (0.4 kW, 20 kHz) for 30 minutes to break aggregates, and then diluted to the exposure concentrations of 100 mg/ml, 75 mg/ml, 50 mg/ml, and 25 mg/ml using micropipettes. These dilutions were then added to the agar wells. Gentamicin was used as a positive control in agar diffusion wells in antimicrobial susceptibility testing. The plates were incubated at 37^p^C for 24 hours and the zones for inhibition that appeared around the wells were recorded. Ampicillin was used as a control for the broth microdilution method to determine the MIC.

MIC of ZrO_2_ NPs against *E. faecalis* and *S. mutans*

MIC is defined as “the lowest concentration of the antimicrobial agent that prevents visible growth of a microorganism under defined conditions” [21]. MIC was determined by the M100 broth microdilution method as per the CLSI guidelines (2017) [22,23].

Inoculum preparation

The procured *E. faecalis *and* S. mutans *were isolated in pure culture. The strains were subcultured onto Brain Heart Infusion (BHI) agar and incubated at 37°C overnight. The inoculum was prepared by suspension of two or more identical colonies in 5 mL of sterile saline. The resulting suspension was vortexed for 15 seconds and the cell density was adjusted with a spectrophotometer by adding sufficient sterile saline to increase the transmittance to that produced by a 0.5 McFarland standard at 530 nm to yield a stock suspension of 1 x 106 to 5 x 106 cells/mL. A working suspension was made by 1:100 dilution of the stock suspension with cation-adjusted Mueller-Hinton broth (cation-adjusted Mueller Hinton broth (CAMHB)) medium, which resulted in 5.0 x 102 to 2.5 x 103 cells/mL.

Procedure

Dilution of ZrO_2_ NPs was performed up to well-10 in a microdilution plate using the broth microdilution method. Of the inoculum suspension in CAMHB, 100μL was added to the series of wells containing ZrO_2_ dilutions. Well-11 was used as a positive media control with only culture media and bacterial strain, and Well-12 was used as a negative growth control with only media. The MIC values were read at 490 nm spectrophotometrically after 24 hours based on the prominent decrease in growth compared to that of the drug-free growth control well.

The powdered ZrO2 NPs used in this study were morphologically and chemically analyzed using SEM and EDAX respectively. Morphological analysis of ZrO2 NPs was done using high-resolution SEM (FEI- Quanta FEG 200F, Sophisticated Analytical Instrument Facility (SAIF), IIT Madras) and elemental analysis was done using EDAX (SAIF, IIT Madras).

## Results

**Table 1:** Calculations for MIC 50 and MIC 90 MIC50: minimum concentration to eradicate approximately 50% of bacterial strain; MIC90: minimum concentration to eradicate approximately 90% of bacterial strain

Bacterial Strain	MIC_50_	MIC_90_
Streptococcus mutans	$\frac{1.307-0.864}{1.307-0.374}\cdot 100 =46\%$	$\frac{1.307-0.436}{1.307-0.374}\cdot 100 = 93\%$
Enterococcus faecalis	$\frac{0.991-0.568}{0.991-0.286}\cdot 100 =60\%$	$\frac{0.991 - 0.568}{0.991 - 0.286}\cdot 100 \leq 60\%$

Antimicrobial susceptibility testing of ZrO_2_ NPs against *E. faecalis* and *S. mutans*

**Figure 1:**
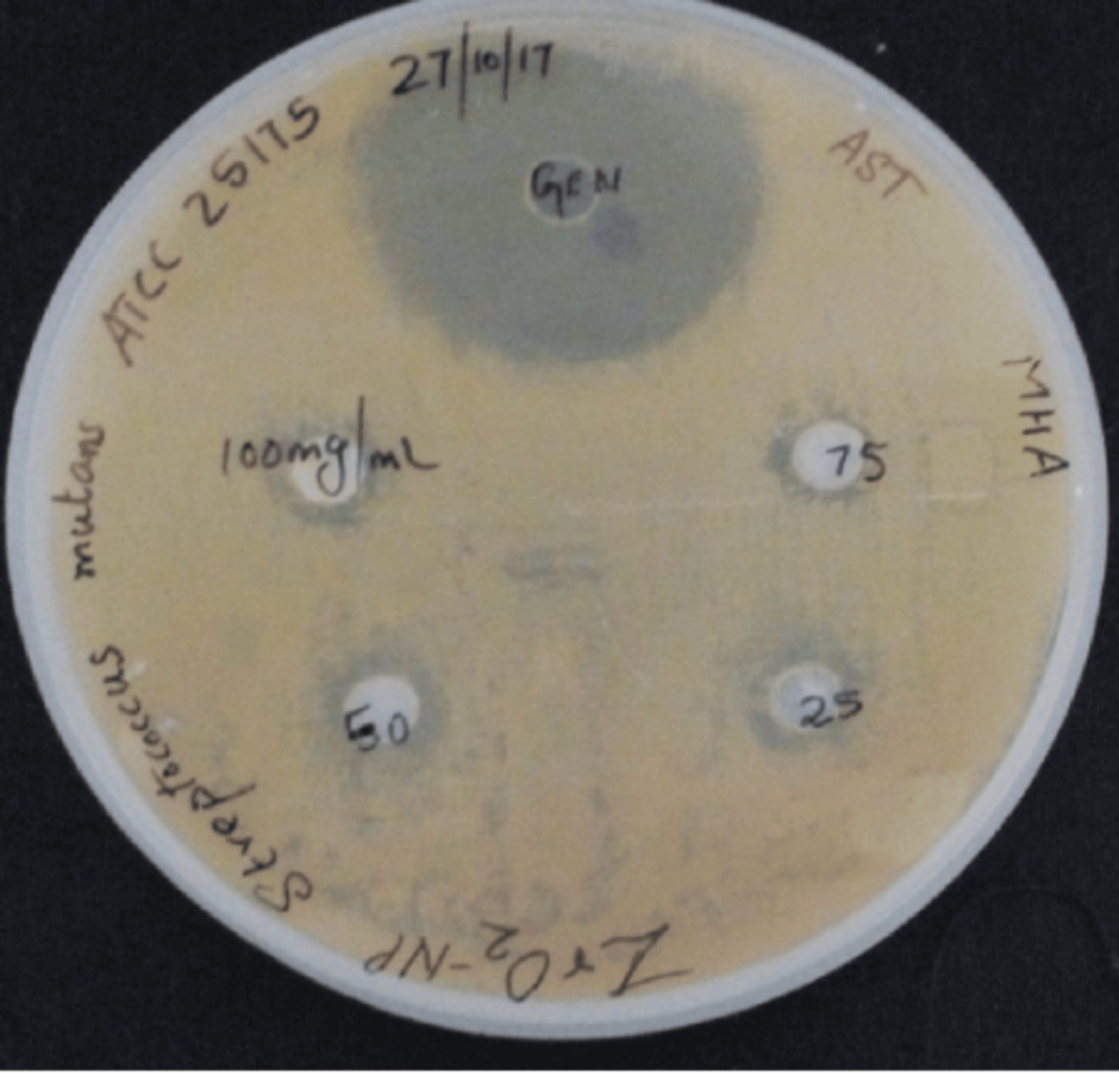
Zone of inhibition of zirconium oxide nanoparticles against Streptococcus mutans ZrO_2_: zirconium oxide; NPs: nanoparticles; MHA: Mueller-Hinton agar; ATCC: American type culture collection; AST: antimicrobial susceptibility testing; Gen: gentamicin

Agar diffusion wells with the selected four dilutions of ZrO_2_ NPs and the positive control of 10 mg/ml gentamicin against *S. mutans* are shown in Figure 1. ZrO2 NPs exhibited antimicrobial properties at 100mg/ml with 12 mm clear zones around the wells. The antimicrobial properties at 25, 50, and 75 mg/ml had a similar diameter of approximately 11 mm of clear zone around the wells.

**Figure 2:**
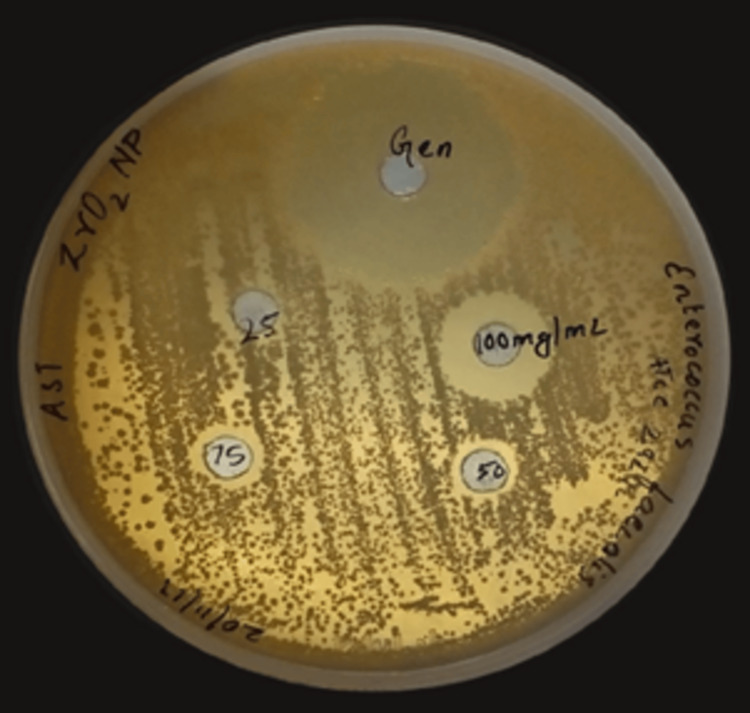
Zone of inhibition of zirconium oxide nanoparticles against Enterococcus faecalis ZrO2: zirconium oxide; NPs: nanoparticles; ATCC: American type culture collection; AST: antimicrobial susceptibility testing; Gen: gentamicin

Similarly, Figure 2 shows that *E. faecalis* is sensitive to ZrO_2_ NPs at a concentration of 100 mg/ml which is evident from the clear zone of 15 mm around the well. However, the zones of inhibition range between 10 mm and 11 mm around the other concentrations of ZrO_2 _NPs against *E.faecalis* (25, 50, and 75 mg/ml).

Gentamicin was used as a positive control against both *S. mutans* and *E. faecalis*, which showed zones of inhibition of 33 mm and 31 mm, respectively. The diameter of the zones of inhibition for various concentrations of ZrO_2_ NPs against *S. mutans* and *E. faecalis* are listed in Table 1.

**Table 2:** Anti-microbial susceptibility testing (Agar well diffusion method) ZrO2: zirconium oxide; NPs: nanoparticles; ATCC: American type culture collection

S. No.	ATCC	Drug Teated (mg/mL)		Zone of Inhibition (diameter in mm)
1.	Streptococcus mutans (25175)	Gentamicin		33
		ZrO_2_ NPs	100	12
			75	11
			50	11
			25	10
2.	Enterococcus faecalis (29212)	Gentamicin		31
		ZrO_2_ NPs	100	15
			75	11
			50	11
			25	10

MIC of ZrO_2_ NPs against *E. faecalis* and *S.mutans*

**Figure 3:**
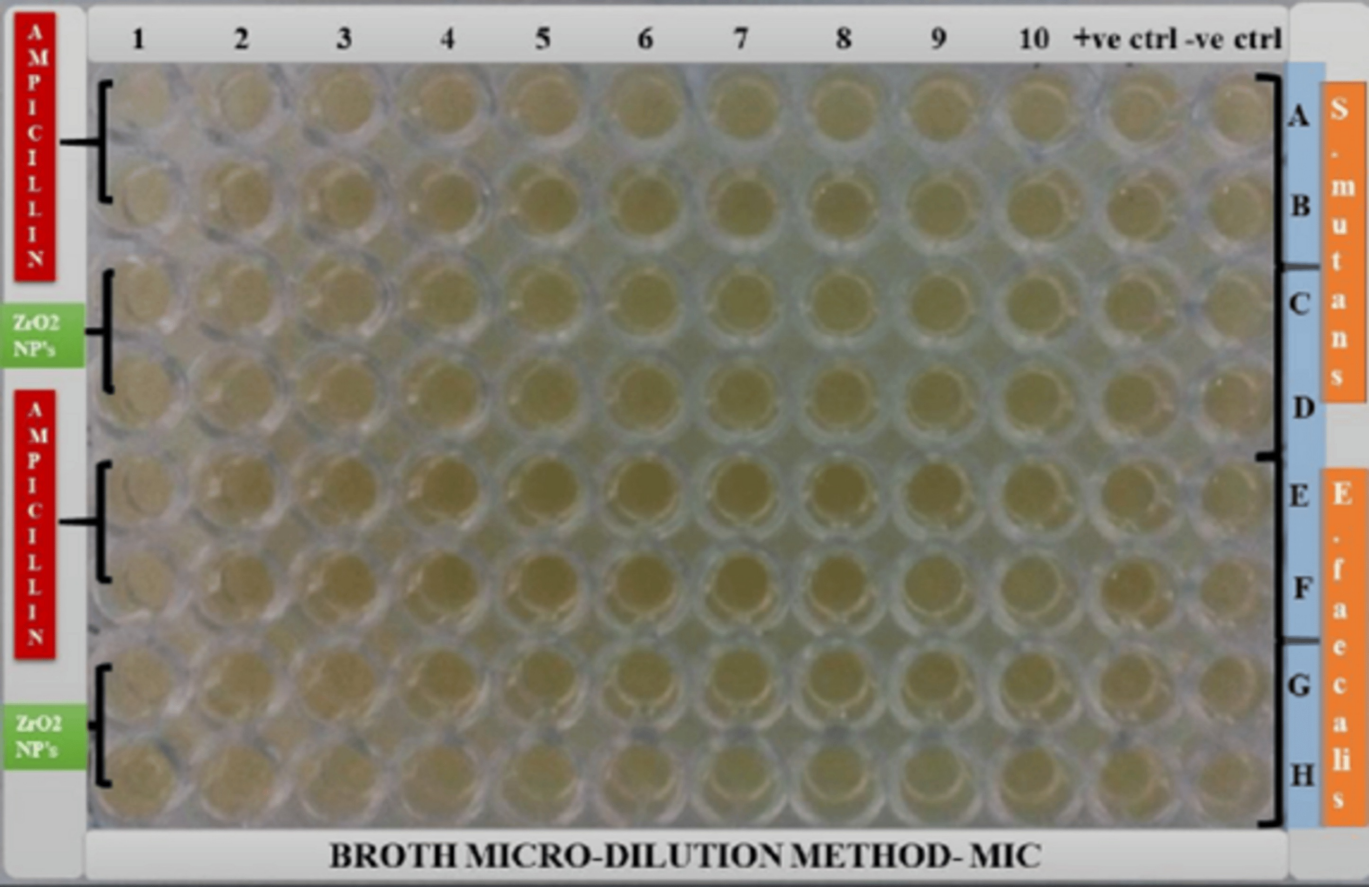
Microtiter plates with serial dilutions of zirconium oxide nanoparticles againist Streptococcus mutans (A,B,C,D) and Enterococcus faecalis (E,F,G,H) Note: Well 11 with positive media control was used with only culture media and bacterial strain, Well 12 with negative media control contains only media with no bacterial strain. ZrO_2_: zirconium oxide; NPs: nanoparticles; MIC: minimum inhibitory concentration

The serial dilutions of ZrO_2_ NPs were performed using the broth M=microdilution method to assess the MIC against *S. mutans* and *E. faecalis* as shown in Figure 3. The microtiter plate was placed in a spectrophotometer to read the optical density values of each well at 490 nm. The spectrophotometer values are presented in Table 2. The obtained values were substituted in the formula to calculate MIC_50 _and MIC_90_ as shown in Table 3. The corresponding dilution of ZrO_2 _NPs at which it eradicated 50% and 90% growth of *S. mutans *and *E. faecalis* is listed in Table 4.

**Table 3:** Spectrometric values (optical density) for minimum inhibitory concentration read at 490 nm Numbers 1-10 signify the well numbers in the Petri dish The letters A-H were used schematically to understand that: Rows A-D had *Streptococcus mutans* strains in which A and B rows were treated with ampicillin as control and C and D rows were treated with Zirconium oxide nanoparticles; Rows E-H had *Enterococcus faecalis* strains in which E and F rows were treated with ampicillin as control and G and H rows were treated with Zirconium oxide nanoparticles ZrO2: zirconium oxide; NPs: nanoparticles

		1	2	3	4	5	6	7	8	9	10	11	12
	A	0.227	0.312	0.286	0.288	0.3	0.291	0.286	0.274	0.286	1.643	1.624	0.392
Ampicillin													
Streptococcus mutans	B	0.237	0.249	0.228	0.23	0.306	0.385	0.294	0.301	0.982	1.779	1.009	0.466
	C	0.436	0.864	0.888	0.863	0.972	0.934	1.115	1.004	1.062	1.053	1.307	0.374
ZrO2 NPs													
	D	0.463	0.369	1.026	1.123	1.144	1.354	1.087	1.062	0.995	0.936	1.099	0.323
	E	0.305	0.294	0.3	0.32	0.316	0.311	0.308	0.295	0.259	1.027	1.557	0.824
Ampicillin													
Enterococcus faecalis	F	0.42	0.334	0.337	0.346	0.332	0.311	0.306	0.294	1.473	1.62	1.205	0.311
	G	0.568	0.943	1.591	1.097	0.836	1.233	1.196	1.108	1.053	1.198	0.991	0.286
ZrO2 NPs													
	H	0.653	1.037	1.444	1.254	1.062	0.986	1.238	1.353	1.199	1.27	0.901	0.281

**Table 4:** Minimum inhibitory concentration (broth dilution method) ATCC: American type culture collection; ZrO2: zirconium oxide; NPs: nanoparticles; MIC: minimum inhibitory concentration; MIC_50_: minimum concentration to eradicate approximately 50% of bacterial strain; MIC_90_: minimum concentration to eradicate approximately 90% of bacterial strain

S.NO	ATCC	Minimum inhibitory concentration			
		Ampicillin		ZrO_2_-NPs	
		MIC 50 (µg/mL)	MIC 90 (µg/mL)	MIC 50 (µg/mL)	MIC 90 (µg/mL)
1.	Streptococcus mutans (25175)	0.5	1	11.4	22.8
2.	Enterococcus faecalis (29212)	0.5	1	22.8	> 22.8

MIC_50 _is the lowest concentration of ZrO_2_ NPs at which 50% of *S. mutans* and *E. faecalis* were inhibited. After substituting the optical density value in the formula, the resultant concentration close to 50% was considered MIC_50 _against both *S. mutans *and* E. faecalis*. This was analyzed to be 11.4mg/ml and 22.8mg/ml for ZrO_2_ NPs against *S. mutans* and *E. faecalis*. Similarly, the MIC that eradicated approximately 90% of bacterial strains was considered to be MIC_90_. This was found to be 22.8 mg/ml and greater for ZrO_2_ NPs against *S. mutans* and *E. faecalis*.

## Discussion

NPs have gained attention in recent years for their inherent antimicrobial properties and affinity to tooth surfaces [24]. NPs have unique physical, chemical, mechanical, magnetic, and electrical characteristics that enable them to enter the cells freely and restrict bacterial metabolism. The properties of NPs that contribute to their efficacy as antimicrobial agents include an increased number of surface atoms, and a higher surface-to-volume ratio [10,25]. This concomitant reduction in particle size along with increased surface area augments the antimicrobial effect by maximizing the area of contact with the bacterial cell [26].

NPs, when used as antimicrobial agents, possess definitive benefits over traditional antibiotics. This is due to the unique physicochemical properties that enable them to overcome drug resistance developed by bacteria against the most common antimicrobial agents [27]. In addition, their reduced size also enables efficient drug delivery, thereby potentiating antimicrobial efficacy [25]. 

ZrO_2_ NPs used in our study were characterized for their physical, chemical, and optical properties using SEM and EDX analysis. The results showed spherical and irregularly spherical ZrO_2_ NPs with an average size of 16.5-27.5nm. Considering that the morphology and size influence antimicrobial activity, this large contact surface enhances the efficacy of ZrO_2_ NPs when compared to their micro-crystalline versions [25,26,28]. These characterized ZrO_2 _NPs were evaluated for their antimicrobial activity against *E. faecalis* and *S. mutans*.

Antimicrobial susceptibility testing

ZrO_2_ NPs showed anti-microbial activity against *S. mutans* and *E. faecalis* at a concentration of 100 mg/mL in the agar diffusion wells. This property can be attributed to the electromagnetic attraction between positively charged zirconium ions from ZrO_2_ NPs, and negatively charged bacterial cell walls, leading to oxidation and death of microorganisms [14]. ZrO_2_ NPs minimize the bacterial adhesion to tooth surfaces by binding to the surface of bacteria. These NPs also inhibit the synthesis of acids, thereby preventing dental caries [14, 28, 29].

The antimicrobial activity of ZrO_2 _NPs has been proposed to result from a few factors. These include the active oxygen species, and slowing of the bacterial growth. Another proposed mechanism is by adversely affecting the integrity of the outer covering of the bacterial cell [30].

MIC

A minimum concentration of 22.8 µg/ml of ZrO_2 _NPs eradicated 90% growth of *S. mutans *and *E. faecalis*, indicating that the antibacterial efficacy of ZrO_2_ NPs is good even at relatively low concentrations. This may enable its usage at concentrations well below its toxicity thresholds. The antimicrobial activity of ZrO_2 _NPs may make these particles beneficial in usage to destroy antibiotic-resistant strains. The anti-microbial efficacy of ZrO_2_ NPs against a few bacterial and fungal strains has also been studied [13,14]. Our current study shows the efficacy of the antibacterial properties of ZrO_2_ NPs against *S. mutans *and *E. faecalis*. To the best of our knowledge and belief, this is new to the published literature. As ZrO_2 _NPs are bactericidal to *S. mutans*, they can be effectively used in formulations for topical antimicrobial oral use, such as dentifrices.

NPs with unique properties such as enhanced surface area and chemical and biological activity can be used as an adjunct to provide complete disinfection of the root canal system. ZrO_2 _NPs having an anti-bacterial activity against E. *faecalis* can be incorporated into irrigant solutions or intracanal medicaments in an attempt to reach the uninstrumented areas at an effective concentration and volume.

Anti-microbial susceptibility testing was used as a screening test for ZrO_2_ NPs against *S. mutans* and* E. faecalis*. Further, quantitative analysis was performed to evaluate the minimum concentration at which anti-bacterial activity occurs. However, there is a need to elucidate the exact mechanism of action of the antibacterial property of ZrO_2 _NPs.

Study limitations and recommendations

Since all the tests were conducted in vitro, it cannot be assumed that the results of antimicrobial efficacy would be proportional or transferable to the oral cavity and translated into clinical effectiveness. In vivo studies are needed. Further studies are recommended, to address the efficacy of ZrO_2 _NPs against cariogenic and endodontic pathogens, to elucidate the interactions of ZrO_2_ NPs in the dental structures, its cytotoxicity, mutagenicity, and other potential long-term effects of ZrO_2_ NPs.

## Conclusions

Within the limitations of this in vitro study, it can be concluded that both *S. mutans* and *E. faecalis* were sensitive to ZrO_2_ NPs at a concentration of 100 mg/mL in the agar well diffusion method of antimicrobial susceptibility testing. The MIC of ZrO_2_ NPs to inhibit 50% growth of *S. mutans* and* E. faecalis *was found to be 11.4 µg/mL and 22.8 µg/mL, respectively, and concentrations higher than 22.8 µg/mL of ZrO_2_ NPs are required to inhibit 90% growth of these two strains.

ZrO_2_ NPs are most widely used in dentistry for improving the physical properties of restorative materials. Identifying the potential antibacterial efficacy against *E. faecalis*, which is the most common organism isolated from secondary endodontic infections can enable the use of ZrO_2_ NPs to be used as an irrigant, medicament, and an additive in endodontic sealer in addition to their desired properties. Similarly, antibacterial efficacy against *S. mutans* can indicate future research incorporating ZrO_2_ NPs in dentifrices and mouth rinses to prevent caries formation. Since the interactions of ZrO_2_ NPs with biosystems are just beginning to be understood, the mechanism of bactericidal actions of ZrO_2_ NPs needs further research.
